# Observation of Gastric Mucosa in Bangladesh, the Country with the Lowest Incidence of Gastric Cancer, and Japan, the Country with the Highest Incidence

**DOI:** 10.1111/j.1523-5378.2012.00967.x

**Published:** 2012-07-02

**Authors:** Takeshi Matsuhisa, Hafeza Aftab

**Affiliations:** * Department of Gastrointestinal Endoscopy, Tama-Nagayama University Hospital of Nippon Medical School1-7-1 Nagayama, Tama-city, Tokyo, 206-8512, Japan; 2 Department of Gastroenterology, Dhaka Medical CollegeDhaka, Bangladesh

**Keywords:** atrophic gastritis, gastric cancer, *Helicobacter pylori* infection, intestinal metaplasia

## Abstract

**Background:**

The prevalence of *Helicobacter pylori* (*H. pylori*) infection is high, but the incidence of gastric cancer is low in natives of Bangladesh. The gastric mucosa was observed in Bangladeshi patients to investigate the differences between Bangladeshis and Japanese.

**Materials and Methods:**

The study involved 418 Bangladeshi and 2356 Japanese patients with abdominal complaints who underwent endoscopy examinations and had no history of *H. pylori* eradication. The prevalence of *H. pylori* infection and the gastric mucosa in *H. pylori*-positive patients were compared between age-, gender-, and endoscopic diagnosis-matched Bangladeshi and Japanese subjects.

**Results:**

The prevalence of *H. pylori* infection was higher in Bangladeshi than in Japanese subjects (60.2 and 45.1%, respectively). All the scores for chronic inflammation, neutrophil activity, glandular atrophy, and intestinal metaplasia were significantly lower in *H. pylori-*positive Bangladeshis than in *H. pylori-*positive Japanese. The ratio of the corpus gastritis score (C) to the antrum gastritis score (A) (C/A ratio) was <1 (antrum-predominant gastritis) in all age groups of Bangladeshi subjects, whereas the C/A ratio changed from <1 to more than 1 (corpus-predominant gastritis) with aging in Japanese subjects.

**Conclusions:**

The scores for glandular atrophy and intestinal metaplasia in *H. pylori-*positive Bangladeshis were significantly lower than those in Japanese. All age groups of Bangladeshis had antrum-predominant gastritis, whereas corpus-predominant gastritis was more common than antrum-predominant gastritis in older Japanese age groups. These results may explain the low incidence of gastric cancer in Bangladeshis and the high incidence in Japanese.

Thirty years have passed since Warren and Marshall discovered *Helicobacter pylori* (*H. pylori*). Researchers now agree that *H. pylori* infection causes not only peptic ulcer disease (PUD) but also gastric cancer 1,2, as revealed by many studies. Japanese men have a high incidence of gastric cancer (46.8 cases per population of 100,000) [Bibr b3]. Despite the high prevalence of *H. pylori* infection in Bangladesh, Thailand, and India, however, the incidence of gastric cancer is extremely low in these countries. These trends have been described as Asian enigmas 4 and Asian paradox 5. Graham et al. 6 subsequently concluded that they are medical myths. In this report, the characteristics of PUD and gastric mucosa were observed and compared in Bangladeshis and Japanese.

## Methods

### Patients

The study involved 418 consecutive outpatients (aged 12–90 years, with a mean age of 36.1 years; male-to-female ratio, 1 : 1.04) who underwent an endoscopy examination at Dhaka Medical College between July 2008 and December 2010 and 2356 consecutive outpatients (aged 11–89 years, with a mean age of 53.0 years; male-to-female ratio, 1 : 0.71) who underwent an endoscopy examination at Nippon Medical School between January 2006 and December 2010. All the patients at both centers had abdominal complaints and no history of having received *H. pylori* eradication therapy. Written informed consent to participate in the study was obtained from all the patients. Because of language differences, the physicians in Bangladesh obtained the written consents from their Bangladeshi patients. In addition, consent for minors to participate in the study was obtained from their legal guardians. All of the cases were examined by the first author using the same criteria.

In total, 415 pairs of 830 patients matched for age (± 5 years), gender and endoscopic diagnosis were used to compare the prevalence of *H. pylori* infection between the two countries, and 212 pairs of 424 *H. pylori-*positive patients from 251 *H. pylori*-positive Bangladeshi and 1280 *H. pylori*-positive Japanese patients matched for age (± 5 years), gender and endoscopic diagnosis were used to compare the gastric mucosa characteristics. In addition, 135 pairs of 270 patients from 251 *H. pylori*-positive and 167 *H. pylori*-negative Bangladeshi patients matched for age (± 5 years), gender and endoscopic diagnosis were used to compare the gastric mucosa between *H. pylori-*positive and -negative Bangladeshis. The endoscopic diagnoses were roughly classified into five categories: gastric cancer, PUD, gastritis (erosion, redness, and/or old bleeding points), normal cases including atrophic gastritis, and other diseases.

### Histological Diagnosis of Gastric Mucosa

**Figure 1:**
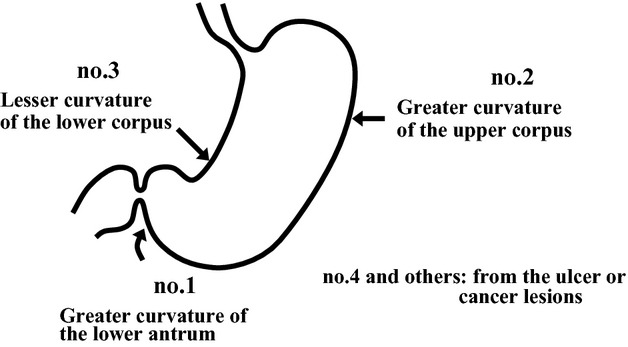
Triple-site biopsy sections are used for the histological diagnosis of chronic inflammation, neutrophil activity, glandular atrophy, intestinal metaplasia, and *Helicobacter pylori* in the gastric mucosa. It was determined between us and a pathologist that section #1 should be taken from the greater curvature of the lower antrum (Antrum), #2 from the greater curvature of the upper corpus (Corpus), and #3 from the lesser curvature of the lower corpus (Angulus). Section #4 and others are taken from the ulcers or cancer lesions.

The gastric mucosa was diagnosed using triple-site biopsy specimens (Fig. 1) 7–11, and chronic inflammation, neutrophil activity, glandular atrophy, intestinal metaplasia, and *H. pylori* were scored using a 4-point scale ranging from 0 to 3, based on the Updated Sydney system (0: none, 1: mild, 2: moderate, and 3: severe). Section #1 was taken from the greater curvature of the lower antrum (Antrum), section #2 was taken from the greater curvature of the upper corpus (Corpus), and section #3 was taken from the lesser curvature of the lower corpus (Angulus). Section #4 and others were taken from the ulcers or cancer lesions. The biopsy sections were stained with hematoxylin–eosin. Giemsa staining was additionally used to diagnose *H. pylori* and immunostaining when it was difficult to assess *H. pylori* infection. When *H. pylori* was detected in at least 1 section, the patient was assessed as *H. pylori* positive.

The ratio of the Corpus activity score (C) (#2, Fig. 1) to the Antrum activity score (A) (#1, Fig. 1), that is, the C/A ratio, was used to diagnose the type of gastritis in *H. pylori-*positive patients 8,9. The C/A ratio in every age group was calculated using the mean score of C divided by the mean score of A. Patients with a C/A ratio of <1 were assessed as having antrum-predominant gastritis and those with a C/A ratio of more than 1 as having corpus-predominant gastritis.

One pathologist (N. Yamada) diagnosed all the sections to minimize any bias in the histological diagnoses.

### Statistical Analysis

The McNemar test was used to compare the prevalence of *H. pylori* infection, and the Mann–Whitney test was used to compare the gastric mucosa, with a significance level of *p* < .05.

## Results

### Prevalence of *Helicobacter pylori* Infection

**Figure 2:**
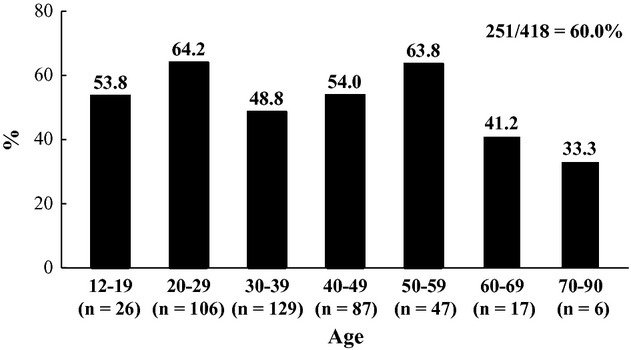
The prevalence of *Helicobacter pylori* infection was 60.0% in all Bangladeshi subjects (418 patients). When compared by age group, the prevalence was highest at ages 20–29, followed by ages 50–59. The prevalence tended to decrease at ages 60 or older (41.2% at ages 60–69, 33.3% at ages 70 or older).

The prevalence of *H. pylori* infection was 60.2% (251 of 418 patients) in Bangladeshis. When compared according to age group, the prevalence was relatively high among young people (19 years or younger: 53.8%, 20–29 years: 64.2%) and tended to decrease in people aged 60 years or older (60–69 years: 41.2%, 70 years or older: 33.3%) (Fig. 2).

The prevalence of *H. pylori* infection was higher in Bangladesh than in Japan (60.2 vs 45.1%, *p* < .0001) (Table 1).

**Table 1:** The prevalence of *Helicobacter pylori* infection between Bangladeshi and Japanese patients -matched for age, gender and endoscopic diagnosis

Prevalence	n	Age (Mean)	Sex ratio
Bangladeshi	415	36.2	203 : 212
Japanese	415	36.1	203 : 212

McNemar's test *p* < .0001.

When compared between Bangladeshi and Japanese patients matched for age, gender, and endoscopic diagnosis, the prevalence of *H. pylori* infection was 60.2% in Bangladeshis and 45.1% in Japanese and was thus significantly higher in Bangladeshis than in Japanese (*p* < .0001).

### Peptic Ulcer Disease

Fifty-one of 418 Bangladeshi patients (12.2%) had PUDs. Out of the PUD patients, 12 had gastric ulcers (GUs; including gastroduodenal ulcers) and 39 had duodenal ulcers (DUs). Thus, the GU/DU ratio was 0.31 (DU predominant). On the other hand, 437 Japanese patients had GU and 269 had DU. Thus, Japanese patients showed a GU predominance, with a GU/DU ratio of 1.62.

### Gastric Mucosa

**Figure 3:**
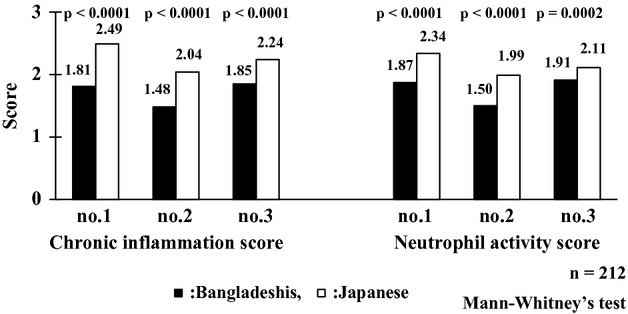
Chronic inflammation, neutrophil activity, glandular atrophy, and intestinal metaplasia scores were compared between *Helicobacter pylori*-positive Bangladeshis and Japanese at each biopsy site, and mean scores in all biopsy sites were significantly higher in Japanese than in Bangladeshis.

The mean scores for chronic inflammation, neutrophil activity, glandular atrophy and intestinal metaplasia for section #1 (Antrum) in the *H. pylori*-positive cases were all significantly lower in Bangladeshis than in Japanese (all *p* < .0001) (Fig. 3). A similar tendency was also seen for sections #2 (Corpus) and #3 (Angulus). (Corpus (Bangladeshi vs Japanese): *p* < .0001, *p* < .0001, *p* < .0001, and *p* = .0138, respectively; Angulus (Bangladeshi vs Japanese): *p* < .0001, *p* = .0002, *p* < .0001, and *p* < .0001, respectively).

**Figure 4:**
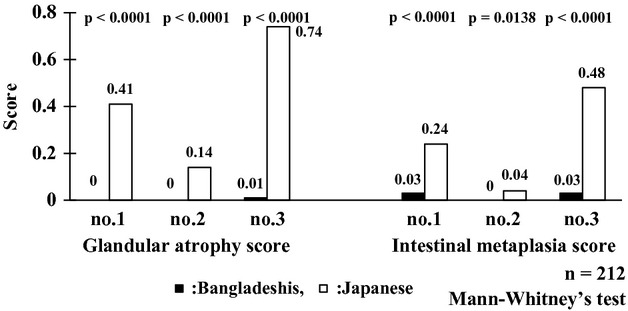
Chronic inflammation and neutrophil activity scores were compared between *Helicobacter pylori*-positive and -negative Bangladeshi subjects in each biopsy site, and mean scores in all biopsy sites were significantly higher in *H. pylori*-positive Bangladeshis. Glandular atrophy and intestinal metaplasia scores were also compared between *H. pylori*-positive and -negative Bangladeshi subjects in each biopsy site, and no differences were found in the mean scores for any biopsy site.

The chronic inflammation and neutrophil activity scores were significantly higher among *H. pylori*-positive Bangladeshis than among *H. pylori*-negative Bangladeshis (chronic inflammation score and neutrophil activity score: Antrum, *p* < .0001 and *p* < .0001; Corpus, *p* < .0001 and *p* < .0001; Angulus, *p* < .0001 and *p* < .0001, respectively) (Fig. 4). However, the glandular atrophy and intestinal metaplasia scores were similar between the *H. pylori*-positive and *H. pylori*-negative Bangladeshis at all sites (mean score of *H. pylori*-positive and *H. pylori*-negative groups: Antrum, 0 and 0.01, respectively; Corpus, 0 and 0, respectively, Angulus, 0 and 0.01, respectively) (Fig. 4).

### C/A Ratio

**Figure 5:**
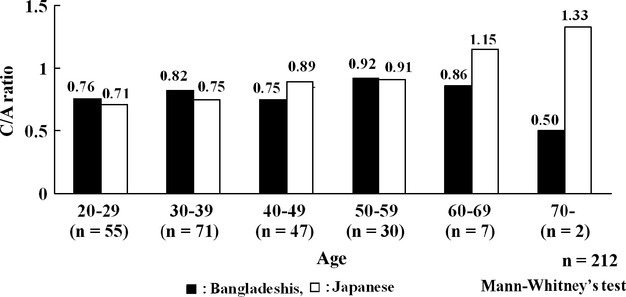
The ratio of the corpus gastritis score to the antrum gastritis score (C/A ratio) was compared between *Helicobacter pylori*-positive Bangladeshi and Japanese patients in each age group. All age groups of Bangladeshis had a C/A ratio of <1 (antrum-predominant gastritis). On the other hand, Japanese aged 59 years or younger had a C/A ratio indicating antrum-predominant gastritis, whereas those aged 60 years or older had a C/A ratio of more than 1, indicating that these Japanese had corpus-predominant gastritis in place of antrum-predominant gastritis.

The C/A ratio in *H. pylori*-positive Bangladeshi showed antrum-predominant gastritis in every age group (Fig. 5). On the other hand, the Japanese exhibited antrum-predominant gastritis in age groups younger than 59 years and corpus-predominant gastritis in age groups older than 60 years (Fig. 5).

### Discussions

According to the age-adjusted numbers of Bangladeshi (male) cancer patients in 2008, the lung was the most common cancer site, followed by the lip/oral cavity, esophagus, other pharynx, and stomach [Bibr b3]. On the other hand, in Japanese men, the stomach was the most common cancer site, followed by the colon/rectum, lung, prostate, and liver [Bibr b3]. Gastric cancer was also the most common cancer in Korean men as well as in Japanese men [Bibr b3]. The age-adjusted mortality rate of gastric cancer has tended to decrease worldwide, and the age-adjusted mortality and incidence rates in Japanese have also tended to decrease 12. However, Asaka et al. 13,14 have pointed out that the number of Japanese patients with gastric cancer will increase with the aging of the population in the future. We investigated differences in the gastric mucosa between Bangladeshis and Japanese from the viewpoint of *H. pylori* infection.

Approximately half of the world's population is said to be infected with *H. pylori*, and the prevalence is higher in developing countries than in developed countries 15. The prevalence is relatively low in the UK, Denmark, and Australia (15% 16, 25.5% 16, and 38% 17, respectively), which are countries that developed earlier, and the *H. pylori*-positive rate is said to be 5% or less in people under 20 years of age and approximately 40% in people in their 50s in these countries 15. The *H. pylori*-positive rate is as high as approximately 50% in people in their teens and more than 90% in people in their 30s in developing countries 18. In Asia, countries such as India, Bangladesh, Pakistan, and Thailand have a high prevalence of *H. pylori* infection. In the presently reported results, the prevalence was as high as 53.8% in Bangladeshis aged 12–19 years, showing a developing country-type prevalence. Although the area surveyed was not clear, a previous report indicated that the prevalence of *H. pylori* infection, as revealed using the serum antibody method, was more than 90% in asymptomatic Bangladeshi adults and 80% in children aged 5 years 19–21. In this study, among all the Bangladeshi patients who visited the hospital with abdominal complaints, the prevalence of *H. pylori* infection, as revealed using microscopic examination, was 60.0% (60.2% in a matched comparison with Japanese patients), which was lower than the previously reported prevalence. The prevalence varied according to the group studied, and it may be possible that the subjects in this study were comprised of people with an average level of living who lived in the central area of the capital, Dhaka, and the prevalence of *H. pylori* infection may be higher in other areas.

In this study, 9.3% of the 418 patients had DUs, and this frequency was similar to that (11.9%) reported by Ahmad et al. 22. in Bangladesh. According to the results of our on-site surveys in Asian countries, the GU/DU ratio was 1.75 (GU-predominant) in Seoul, Korea (data not shown). Wong et al. 23 compared four areas in northern China and four areas in southern China and reported that the DU/GU ratio was lower in northern China (1.3) than in southern China (2.41) (0.77 and 0.40, respectively, when the reported values were converted to a GU/DU ratio). Thus, considerable differences exist among Asian countries in that patients in Japan and Korea exhibit a GU predominance while those in other countries, including Bangladesh, exhibit a DU predominance. Concerning the relationship between PUD and gastric cancer, Chiba 24 has described that the higher the GU/DU ratio in a country or region, the higher the incidence of gastric cancer. The development of gastric cancer is also positively correlated with GUs but negatively correlated with DUs 25. Based on these observations, one may presume that gastric cancer is common among Japanese but not among Bangladeshis.

The scores for chronic inflammation, neutrophil activity, glandular atrophy, and intestinal metaplasia were significantly lower in *H. pylori*-positive Bangladeshis than in Japanese at all the gastric sites that were examined. Western-type and East Asian-type strains of *H. pylori* are known to exist. People are infected with East Asian-type and Western-type *H. pylori* strains in countries east and west of Thailand, respectively, and Bangladeshis are typically infected with Western-type *H. pylori* strains 26. East Asian-type *H. pylori* strains cause more intense chronic inflammation and neutrophil activity than Western-type *H. pylori* strains 27, and East Asian-type *H. pylori* strains are involved in gastric mucosal atrophy and gastric cancer 28. The scores for glandular atrophy and intestinal metaplasia were almost zero even among *H. pylori*-positive Bangladeshi patients, indicating that Western-type *H. pylori* strains do not induce glandular atrophy or intestinal metaplasia. In our previously reported results, these scores were also significantly lower in Nepalese than in Japanese 9. We previously conducted a survey in Chiang Mai, an area where the incidence of gastric cancer is highest in Thailand 29, and the scores for glandular atrophy and intestinal metaplasia were also significantly lower in Chiang Mai than in Japan 7. Uemura et al. 1 have reported that the presence of atrophic gastritis and intestinal metaplasia is strongly involved in the risk of gastric cancer and that severe atrophic gastritis with intestinal metaplasia, in particular, is associated with a high risk of differentiated gastric cancer.

All age groups of Bangladeshi subjects had antrum-predominant gastritis, similar to the Thais 8 and Nepalese 9. On the other hand, with aging, the Japanese tend to develop corpus-predominant gastritis, rather than antrum-predominant gastritis. The risk of gastric cancer is reportedly 23.3 times higher for corpus-predominant gastritis than for antrum-predominant gastritis 1, consistent with the low incidence of gastric cancer in Bangladeshis and the high incidence in Japanese. Graham hypothesized that poor nutrition during childhood in *H. pylori*-infected individuals leads to low acid secretion, and low acid secretion accompanied by infection with a highly virulent strain of *H. pylori* (cagA-positive) leads to progressive and multifocal atrophic gastritis and increases the risk of gastric cancer 30. Our results prove this hypothesis.

According to the guidelines of the Japanese Society for Helicobacter Research published in 2009, eradication therapy should be given to all patients with *H. pylori* infection 31. According to a report by Uemura et al. 1, gastric cancer occurred only in *H. pylori*-positive patients with functional dyspepsia, GUs, and gastric polyps, but not in *H. pylori*-positive patients with DUs, over the observation period of their study (mean, 7.8 years; maximum, 10.6 years). In addition, Take et al. 32 reported that gastric cancer occurred only in GU patients, but not in DU patients, during the follow-up of PUD patients after eradication therapy (mean, 3.4 years; maximum, 8.6 years). Considering these reports, eradication therapy to prevent PUD recurrence, rather than to prevent gastric cancer, seems to be sufficient in Bangladeshi patients.

In conclusion, a comparative observation of PUD and the gastric mucosa was conducted in Bangladeshis and Japanese. As a result, the prevalence of *H. pylori* infection was found to be higher in Bangladeshis than in Japanese, particularly in young Bangladeshis aged 29 years or less. PUD was DU predominant in Bangladeshis and GU predominant in Japanese. The scores for glandular atrophy and intestinal metaplasia were lower in Bangladeshis than in Japanese, and these scores were remarkably low in *H. pylori*-positive Bangladeshis, with no difference between *H. pylori*-positive and *H. pylori*-negative Bangladeshi subjects. All the age groups of *H. pylori*-positive Bangladeshis had antrum-predominant gastritis, whereas the *H. pylori*-positive Japanese developed corpus-predominant gastritis with aging, rather than antrum-predominant gastritis. These differences seemed to affect and may possibly explain the low incidence of gastric cancer in Bangladeshis and the high incidence in Japanese.
